# A co-crystal of nona­hydrated disodium(II) with mixed anions from *m*-chloro­benzoic acid and furosemide

**DOI:** 10.1107/S2056989015017430

**Published:** 2015-09-30

**Authors:** Bianca King London, Michelle O. Fletcher Claville, Sainath Babu, Frank R. Fronczek, Rao M. Uppu

**Affiliations:** a Environmental Toxicology PhD Program and the Health Research Center, Southern University and A&M College, Baton Rouge, LA 70813, USA; b School of Science, Hampton University, Hampton, VA 23668, USA; c Department of Chemistry, Louisiana State University, Baton Rouge, LA 70803-1804, USA

**Keywords:** crystal structure, loop diuretics, co-crystals, pharmaceutical formulations, hydrogen bonding

## Abstract

In the title compound, [Na_2_(H_2_O)_9_](C_7_H_4_ClO_2_)(C_12_H_10_ClN_2_O_5_S) {systematic name: *catena*-poly[[[triaquasodium(I)]-di-μ-aqua-[triaquasodium(I)]-μ-aqua] 3-chlorobenzoate 4-chloro-2-[(furan-2-ylmethyl)amino]-5-sulfamoylbenzoate]}, both the original *m*-chloro­benzoic acid and furosemide exist with deprotonated carboxyl­ates, and the sodium cations and water mol­ecules exist in chains with stoichiometry [Na_2_(OH_2_)_9_]^2+^ that propagate in the [-110] direction. Each of the two independent Na^+^ ions is coordinated by three monodentate water mol­ecules, two double-water bridges, and one single-water bridge. There is considerable cross-linking between the [Na_2_(OH_2_)_9_]^2+^ chains and to furosemide sulfonamide and carboxyl­ate by inter­molecular O—H⋯O hydrogen bonds. All hydrogen-bond donors participate in a complex two-dimensional array parallel to the *ab* plane. The furosemide NH group donates an intra­molecular hydrogen bond to the carboxyl­ate group, and the furosemide NH_2_ group donates an intra­molecular hydrogen bond to the Cl atom and an inter­molecular one to the *m*-chloro­benzoate O atom. The plethora of hydrogen-bond donors on the cation/water chain leads to many large rings, up to graph set *R*_4_^4^(24), involving two chains and two furosemide anions. The chloro­benzoate is involved in only one *R*_2_^2^(8) ring, with two water mol­ecules *cis*-coordinated to Na. The furan O atom is not hydrogen bonded.

## Abstract 2

In view of its potential for developing useful pharamaceutical formulations of furosemide, a widely-used loop diuretic, the crystal structure of the furosemide anion with *m*-chloro­benzoate has been investigated. In these co-crystals, the monoanions of furosemide and *m*-chloro­benzoate are balanced by two independent Na^+^ ions, both of which are hexa­coordinated by three monodentate water mol­ecules, two double-water bridge mol­ecules and one single-water bridge mol­ecules, thus yielding centrosymmetric Na_2_(OH_2_)_8_ units linked by single water bridges to form chains in the [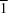
10] direction.

## Chemical context   

Furosemide is a widely used diuretic for the treatment of hypertension and edema (Krumlovsky & del Greco, 1976 ▸; Musini *et al.*, 2015 ▸), and to a lesser extent, hypercalcemia (Belen *et al.*, 2014 ▸; Carvalhana *et al.*, 2006 ▸). While this furan-containing compound is of inter­est, the toxicity elicited by these core compounds is not well understood. The free furan itself is a known hepato-carcinogen and toxicant, as studied in rats (Gill *et al.*, 2010 ▸) and mice (Terrell *et al.*, 2014 ▸). The epoxide metabolite of furans, formed in CYP450-mediated oxidations, can isomerize to highly reactive electro­philic inter­mediates such as *cis*-2-butene-1,4-dial (Chen *et al.*, 1995 ▸; Peterson 2015 ▸; Vargas *et al.*, 1998 ▸).

We have performed the oxidation of furosemide with *m*-chloro­perbenzoic acid (*m*-CPBA), and isolated various epoxide and isomerized products in support of our efforts to understand this type of toxicity mechanism, and to also identify potential biomarkers for furosemide in humans. During the separation and drying of the products of the furosemide–*m*-CPBA reaction, we observed the formation of crystals in the mother liquor (the organic layer). Analysis of these crystals by X-ray crystallography revealed a disodium nona­hydrate co-crystal with furosemide (starting material) and *m*-chloro­benzoic acid (an inadvertent contaminant or the reduced product of *m*-CPBA). Analogous to the known properties of co-crystals of furosemide with nicotinamide and their pharmaceutical importance (Aitipamula *et al.*, 2012 ▸; Chadha *et al.*, 2012 ▸; Goud *et al.*, 2012 ▸; Stepanovs & Mishnev, 2012 ▸; Ueto *et al.*, 2012 ▸), we believe that the co-crystals of furosemide with *m*-chloro­benzoic acid could have useful applications in drug development and may lead to formulations with improved potency, solubility, and stability. Therefore, this serendipitous finding may have important applications for improving furosemide bioavailability.
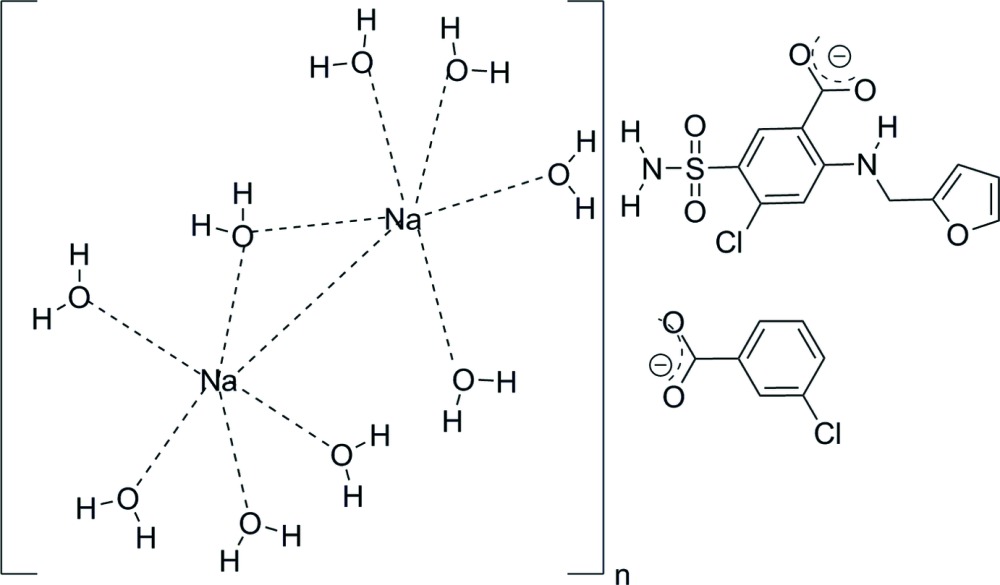


## Structural commentary   

**Figure 1:**
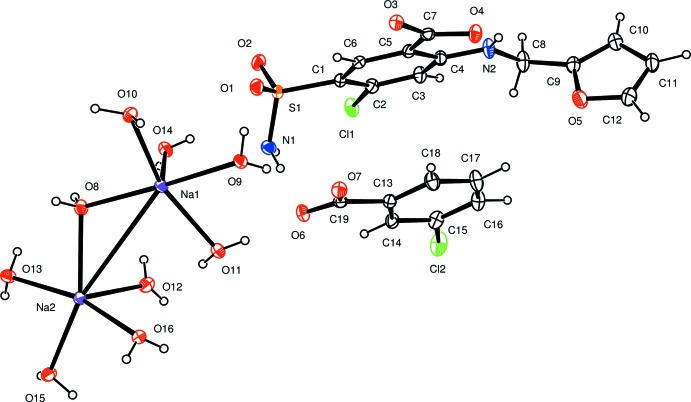
The asymmetric unit with 50% ellipsoids.

**Figure 2:**
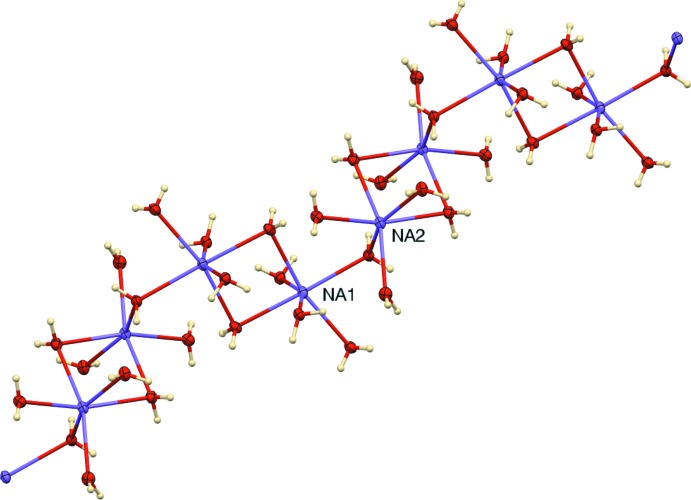
A portion of the Na–water chain, showing the centrosymmetric Na_2_(OH_2_)_2_ bridges and the single water bridges.

The asymmetric unit is illustrated in Fig. 1 ▸. The furosemide moiety is present as the monoanion, with the COOH group deprotonated, N2 as NH and the primary amine nitro­gen N1 as NH_2_. The *m*-chlorobenzoic acid moiety is also deprotonated. Balancing the charge of the two types of anions are two independent sodium cations, both of which are hexa­coordinate, with Na⋯O(water) distances in the range 2.3558 (13)–2.4500 (13) Å. Each Na^+^ cation is coordinated by three monodentate water mol­ecules, two double-water bridge mol­ecules, and one single-water bridge mol­ecule, as shown in Fig. 2 ▸. Thus, centrosymmetric Na_2_(OH_2_)_8_ units are linked by single water bridges, forming chains in the [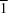
10] direction.

## Supra­molecular features   

**Figure 3:**
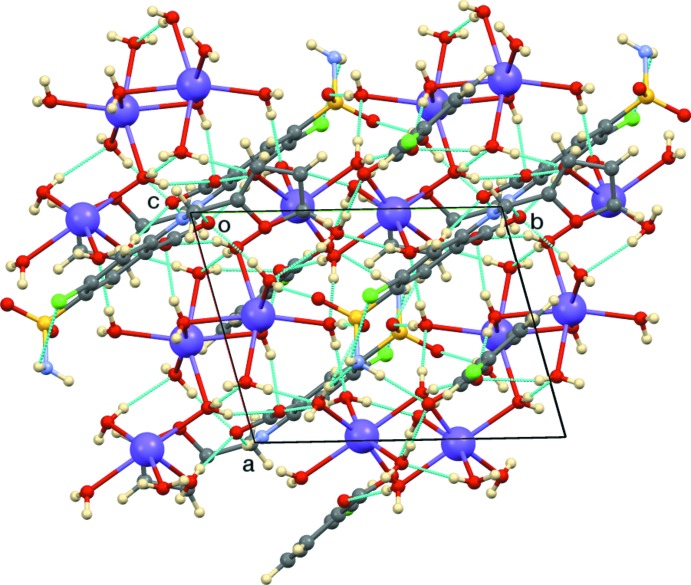
A supra­molecular layer of the title compound in the *ab* plane.

Hydrogen bonding is extensive (Table 1 ▸), with all 21 hydrogen-bond donors involved. Notable features of the two-dimensional hydrogen-bonding pattern (Etter *et al.*. 1990 ▸) are sulfonamide N—H⋯O bonds to *m*-chloro­benzoate, secondary amine N—H⋯O hydrogen bonds to furosemide anion (carboxyl­ate), and water O—H⋯O hydrogen bonds to the sulfona­mide O atom, to both types of carboxyl­ates, and to other water mol­ecules. The direction of the normal to the hydrogen-bonding network is [001]. The furan oxygen atom O5 is not involved in the hydrogen bonding. A supramolecular layer in the ab plane is shown in Fig. 3 ▸.

## Synthesis and crystallization   

**Figure 4:**
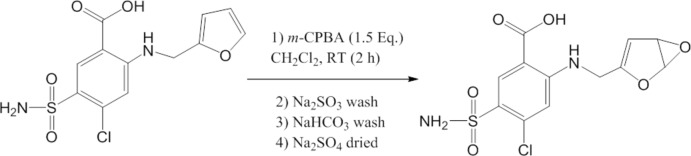
Proposed scheme of reactions of furosemide with *m*-chloro­per­oxy­benzoic acid.

Furosemide (8.2 mmol; 2.71 g), dissolved in 3 ml of di­chloro­methane (DCM), was added dropwise over 5 min to a solution of 8.2 mmol of *m*-CPBA (1.84 g) and 10.5 mmol NaHCO_3_ (0.88 g) in 20 ml of DCM on ice with rapid stirring (Fig. 4 ▸). After 2 h, an additional 4 mmol of *m*-CPBA in 10 ml of DCM was added to the reaction mixture. Upon removal from the ice bath, 4 ml of aqueous sodium sulfite solution (10%) was added with stirring for an additional 15 min. After partitioning the layers with deionized water (resistance 18.2 *M* Ω cm^−1^), the organic layer was collected and the aqueous layer was extracted with another 10 ml of DCM. The combined mixture of the organic layer was washed with 10 ml of aqueous solution of NaHCO_3_ (5%, *w*/*v*), dried over anhydrous Na_2_SO_4_, and then subjected to partial evaporation under low pressure (*ca* 4 psi) at 308 K. The partially evaporated sample was left at ambient pressure and temperature overnight. Crystals were formed with slow evaporation.

## Refinement   

Crystal data, data collection and structure refinement details are summarized in Table 2 ▸. H atoms on C were idealized with C—H distances of 0.95 Å for *sp* ^2^ C and 0.99 Å for CH_2_. Those on N and O were assigned from difference maps, and their positions refined, with O—H distances restrained to be equal. *U*_iso_(H) were assigned as 1.2 times *U*_eq_ of the attached atoms (1.5 for water). Six reflections with *F*o<<*F*c were omitted from the calculations.

## Supplementary Material

Crystal structure: contains datablock(s) New_Global_Publ_Block, I. DOI: 10.1107/S2056989015017430/pk2557sup1.cif

Structure factors: contains datablock(s) I. DOI: 10.1107/S2056989015017430/pk2557Isup2.hkl

CCDC reference: 1425658

Additional supporting information:  crystallographic information; 3D view; checkCIF report

## Figures and Tables

**Table 1:** Hydrogen-bond geometry (, )

*D*H*A*	*D*H	H*A*	*D* *A*	*D*H*A*
N1H11*N*Cl1	0.839(18)	2.767(17)	3.2848(16)	121.6(14)
N1H12*N*O6	0.872(18)	1.920(18)	2.7905(17)	175.3(17)
N2H2*N*O4	0.860(18)	1.906(18)	2.6199(16)	139.4(16)
O8H81O3^i^	0.83(2)	2.07(2)	2.8737(16)	162(2)
O8H82O15^ii^	0.82(2)	2.00(2)	2.8025(16)	168(2)
O9H91O16^iii^	0.86(2)	1.99(2)	2.8499(15)	172(2)
O9H92O7	0.81(2)	2.10(2)	2.8867(16)	167(2)
O10H101O11^iii^	0.78(2)	1.96(2)	2.7444(16)	174(2)
O10H102O3^iv^	0.93(2)	1.90(2)	2.8271(15)	171(2)
O11H111O6	0.84(2)	1.89(2)	2.7296(16)	176(2)
O11H112O12	0.81(2)	2.08(2)	2.8641(16)	162(2)
O12H121O4^i^	0.85(2)	1.96(2)	2.7962(16)	171(2)
O12H122O2^v^	0.76(2)	2.12(2)	2.8593(15)	165(2)
O13H131O7^i^	0.81(2)	2.01(2)	2.8082(15)	169(2)
O13H132O3^i^	0.85(2)	1.97(2)	2.7939(15)	165(2)
O14H141O4^i^	0.76(2)	1.99(2)	2.7265(16)	164(2)
O14H142O1	0.74(2)	2.31(2)	3.0019(17)	156(2)
O15H151O14^v^	0.85(2)	1.92(2)	2.7766(16)	178(2)
O15H152O7^i^	0.80(2)	2.14(2)	2.8545(16)	149(2)
O16H161O10^v^	0.798(19)	1.990(19)	2.7857(16)	175.7(18)
O16H162O1^v^	0.80(2)	2.20(2)	2.9852(15)	165.8(17)

Symmetry codes: (i) 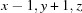; (ii) 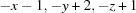; (iii) 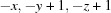; (iv) 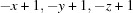; (v) 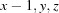.

**Table 2:** Experimental details

Crystal data	
Chemical formula	[Na_2_(OH_2_)_9_](C_7_H_4_ClO_2_)(C_12_H_10_ClN_2_O_5_S)
*M*_r_	693.41
Crystal system, space group	Triclinic, *P* 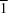
Temperature (K)	100
*a*, *b*, *c* ()	7.908(2), 10.224(3), 19.631(4)
, , ()	85.46(2), 81.80(2), 74.96(2)
*V* (^3^)	1515.7(7)
*Z*	2
Radiation type	Mo *K*
(mm^1^)	0.39
Crystal size (mm)	0.30 0.25 0.07

Data collection	
Diffractometer	Nonius KappaCCD
Absorption correction	Multi-scan (*SCALEPACK*; Otwinowski Minor, 1997 ▸)
*T*_min_, *T*_max_	0.893, 0.974
No. of measured, independent and observed [*I* > 2s(*I*)] reflections	17752, 10267, 7760
*R*_int_	0.035
(sin /)_max_ (^1^)	0.751

Refinement	
*R*[*F* ^2^ > 2(*F* ^2^)], *wR*(*F* ^2^), *S*	0.043, 0.107, 1.05
No. of reflections	10267
No. of parameters	442
No. of restraints	120
H-atom treatment	H atoms treated by a mixture of independent and constrained refinement
_max_, _min_ (e ^3^)	0.47, 0.55

Computer programs: *COLLECT* (Nonius, 1999 ▸), *HKL* *SCALEPACK* and *DENZO* and *SCALEPACK* (Otwinowski Minor 1997 ▸), *SIR97* (Altomare *et al.*, 1999 ▸), *SHELXL97* (Sheldrick, 2008 ▸) and *ORTEP-3 for Windows* (Farrugia, 2012 ▸).

## supplementary crystallographic information

### Crystal data

**Table:**

[Na_2_(OH_2_)_9_](C_7_H_4_ClO_2_)(C_12_H_10_ClN_2_O_5_S)	*Z* = 2
*M**_r_* = 693.41	*F*(000) = 720
Triclinic, *P*1	*D*_x_ = 1.519 Mg m^−^^3^
Hall symbol: -P 1	Mo *K*α radiation, λ = 0.71073 Å
*a* = 7.908 (2) Å	Cell parameters from 17752 reflections
*b* = 10.224 (3) Å	θ = 2.5–32.6°
*c* = 19.631 (4) Å	µ = 0.39 mm^−^^1^
α = 85.46 (2)°	*T* = 100 K
β = 81.80 (2)°	Lath fragment, colorless
γ = 74.96 (2)°	0.30 × 0.25 × 0.07 mm
*V* = 1515.7 (7) Å^3^	

### Data collection

**Table:**

Nonius KappaCCD diffractometer	10267 independent reflections
Radiation source: fine-focus sealed tube	7760 reflections with *I* > 2s(*I*)
Graphite monochromator	*R*_int_ = 0.035
ω and φ scans	θ_max_ = 32.3°, θ_min_ = 2.7°
Absorption correction: multi-scan (*SCALEPACK*; Otwinowski & Minor, 1997)	*h* = −11→11
*T*_min_ = 0.893, *T*_max_ = 0.974	*k* = −13→15
17752 measured reflections	*l* = −29→29

### Refinement

**Table:**

Refinement on *F*^2^	Primary atom site location: structure-invariant direct methods
Least-squares matrix: full	Secondary atom site location: difference Fourier map
*R*[*F*^2^ > 2σ(*F*^2^)] = 0.043	Hydrogen site location: inferred from neighbouring sites
*wR*(*F*^2^) = 0.107	H atoms treated by a mixture of independent and constrained refinement
*S* = 1.05	*w* = 1/[σ^2^(*F*_o_^2^) + (0.058*P*)^2^] where *P* = (*F*_o_^2^ + 2*F*_c_^2^)/3
10267 reflections	(Δ/σ)_max_ = 0.001
442 parameters	Δρ_max_ = 0.47 e Å^−^^3^
120 restraints	Δρ_min_ = −0.55 e Å^−^^3^

### Special details

**Table:**

Geometry. All e.s.d.'s (except the e.s.d. in the dihedral angle between two l.s. planes) are estimated using the full covariance matrix. The cell e.s.d.'s are taken into account individually in the estimation of e.s.d.'s in distances, angles and torsion angles; correlations between e.s.d.'s in cell parameters are only used when they are defined by crystal symmetry. An approximate (isotropic) treatment of cell e.s.d.'s is used for estimating e.s.d.'s involving l.s. planes.
Refinement. Refinement of *F*^2^ against ALL reflections. The weighted *R*-factor *wR* and goodness of fit *S* are based on *F*^2^, conventional *R*-factors *R* are based on *F*, with *F* set to zero for negative *F*^2^. The threshold expression of *F*^2^ > σ(*F*^2^) is used only for calculating *R*-factors(gt) *etc*. and is not relevant to the choice of reflections for refinement. *R*-factors based on *F*^2^ are statistically about twice as large as those based on *F*, and *R*- factors based on ALL data will be even larger.

### Fractional atomic coordinates and isotropic or equivalent isotropic displacement parameters (Å^2^)

**Table:**

	*x*	*y*	*z*	*U*_iso_*/*U*_eq_	
Cl1	0.62548 (4)	0.49926 (3)	0.089838 (16)	0.01988 (8)	
S1	0.53103 (4)	0.56412 (3)	0.252718 (16)	0.01360 (7)	
O1	0.49898 (12)	0.54858 (10)	0.32676 (5)	0.01772 (19)	
O2	0.61708 (13)	0.66762 (9)	0.22345 (5)	0.0186 (2)	
O3	0.86565 (12)	0.10830 (9)	0.36913 (5)	0.01511 (18)	
O4	0.95416 (13)	−0.04844 (9)	0.29000 (5)	0.01778 (19)	
O5	0.92982 (13)	−0.22550 (10)	0.12326 (6)	0.0250 (2)	
N1	0.34022 (15)	0.59988 (12)	0.22586 (6)	0.0171 (2)	
H11N	0.345 (2)	0.6226 (18)	0.1837 (10)	0.021*	
H12N	0.282 (2)	0.5394 (18)	0.2404 (9)	0.021*	
N2	0.97465 (16)	0.03032 (12)	0.15946 (6)	0.0191 (2)	
H2N	1.009 (2)	−0.0258 (18)	0.1927 (9)	0.023*	
C1	0.65991 (16)	0.40639 (13)	0.22332 (6)	0.0133 (2)	
C2	0.70427 (17)	0.37919 (13)	0.15334 (7)	0.0149 (2)	
C3	0.81105 (18)	0.25674 (13)	0.13183 (7)	0.0165 (2)	
H3	0.8422	0.2422	0.0840	0.020*	
C4	0.87486 (17)	0.15226 (13)	0.18053 (7)	0.0147 (2)	
C5	0.83234 (16)	0.17957 (12)	0.25209 (6)	0.0128 (2)	
C6	0.72692 (16)	0.30566 (13)	0.27139 (7)	0.0131 (2)	
H6	0.6996	0.3237	0.3190	0.016*	
C7	0.88889 (16)	0.07359 (13)	0.30778 (7)	0.0133 (2)	
C8	1.0003 (2)	−0.01178 (14)	0.08920 (7)	0.0215 (3)	
H8A	1.0944	0.0249	0.0619	0.026*	
H8B	0.8900	0.0247	0.0682	0.026*	
C9	1.05072 (18)	−0.16262 (14)	0.08795 (7)	0.0179 (3)	
C10	1.19245 (19)	−0.25494 (14)	0.05907 (7)	0.0193 (3)	
H10	1.2949	−0.2372	0.0325	0.023*	
C11	1.1573 (2)	−0.38525 (14)	0.07659 (7)	0.0211 (3)	
H11	1.2312	−0.4709	0.0634	0.025*	
C12	0.9991 (2)	−0.36173 (15)	0.11544 (9)	0.0252 (3)	
H12	0.9430	−0.4302	0.1348	0.030*	
Cl2	0.28737 (6)	0.23829 (4)	0.026181 (19)	0.02904 (9)	
O6	0.15314 (12)	0.41020 (9)	0.27976 (5)	0.01757 (19)	
O7	0.25838 (13)	0.23500 (10)	0.35073 (5)	0.01731 (19)	
C13	0.31680 (17)	0.20440 (13)	0.22941 (7)	0.0147 (2)	
C14	0.27177 (18)	0.25472 (13)	0.16425 (7)	0.0162 (2)	
H14	0.1917	0.3408	0.1589	0.019*	
C15	0.34577 (19)	0.17705 (14)	0.10766 (7)	0.0191 (3)	
C16	0.4648 (2)	0.05117 (14)	0.11350 (8)	0.0230 (3)	
H16	0.5148	−0.0002	0.0740	0.028*	
C17	0.5091 (2)	0.00209 (15)	0.17849 (8)	0.0243 (3)	
H17	0.5903	−0.0836	0.1836	0.029*	
C18	0.43507 (18)	0.07803 (14)	0.23614 (7)	0.0198 (3)	
H18	0.4654	0.0434	0.2804	0.024*	
C19	0.23698 (16)	0.28915 (13)	0.29148 (7)	0.0140 (2)	
Na1	0.01996 (6)	0.65083 (5)	0.45870 (3)	0.01372 (11)	
Na2	−0.44798 (7)	0.87445 (5)	0.44265 (3)	0.01394 (11)	
O8	−0.15551 (12)	0.88218 (10)	0.46379 (5)	0.01546 (18)	
H81	−0.126 (2)	0.9400 (19)	0.4362 (10)	0.023*	
H82	−0.175 (2)	0.9155 (18)	0.5017 (10)	0.023*	
O9	0.19980 (12)	0.42119 (10)	0.45986 (5)	0.01525 (18)	
H91	0.295 (2)	0.4001 (18)	0.4793 (9)	0.023*	
H92	0.217 (2)	0.3797 (19)	0.4253 (10)	0.023*	
O10	0.18912 (12)	0.66647 (10)	0.54828 (5)	0.01560 (18)	
H101	0.169 (2)	0.6053 (19)	0.5718 (10)	0.023*	
H102	0.158 (2)	0.7401 (18)	0.5767 (9)	0.023*	
O11	−0.10007 (13)	0.54861 (10)	0.37594 (5)	0.01550 (18)	
H111	−0.026 (2)	0.5051 (18)	0.3452 (10)	0.023*	
H112	−0.161 (2)	0.6123 (19)	0.3560 (9)	0.023*	
O12	−0.30234 (14)	0.80747 (10)	0.33026 (5)	0.0182 (2)	
H121	−0.221 (2)	0.8455 (19)	0.3142 (10)	0.027*	
H122	−0.329 (2)	0.7843 (19)	0.2985 (10)	0.027*	
O13	−0.47562 (13)	1.11930 (10)	0.43409 (5)	0.01560 (18)	
H131	−0.543 (2)	1.1568 (19)	0.4070 (10)	0.023*	
H132	−0.380 (2)	1.1303 (18)	0.4114 (10)	0.023*	
O14	0.16422 (13)	0.76011 (10)	0.36697 (5)	0.01745 (19)	
H141	0.108 (3)	0.802 (2)	0.3408 (10)	0.026*	
H142	0.236 (3)	0.713 (2)	0.3462 (10)	0.026*	
O15	−0.73738 (13)	0.97411 (10)	0.41518 (5)	0.01661 (19)	
H151	−0.770 (2)	0.9090 (19)	0.4010 (9)	0.025*	
H152	−0.753 (2)	1.0342 (19)	0.3861 (10)	0.025*	
O16	−0.49332 (13)	0.65364 (10)	0.46360 (5)	0.01549 (18)	
H161	−0.582 (2)	0.6532 (18)	0.4885 (10)	0.023*	
H162	−0.494 (2)	0.6123 (18)	0.4305 (10)	0.023*	

### Atomic displacement parameters (Å^2^)

**Table:**

	*U*^11^	*U*^22^	*U*^33^	*U*^12^	*U*^13^	*U*^23^
Cl1	0.02539 (17)	0.01556 (15)	0.01459 (14)	0.00207 (12)	−0.00343 (12)	0.00125 (11)
S1	0.01423 (14)	0.01061 (14)	0.01548 (15)	−0.00184 (11)	−0.00167 (11)	−0.00254 (11)
O1	0.0191 (5)	0.0175 (5)	0.0155 (4)	−0.0020 (4)	−0.0016 (4)	−0.0041 (4)
O2	0.0209 (5)	0.0136 (4)	0.0221 (5)	−0.0062 (4)	−0.0016 (4)	−0.0022 (4)
O3	0.0167 (4)	0.0147 (4)	0.0138 (4)	−0.0036 (4)	−0.0024 (3)	−0.0002 (3)
O4	0.0240 (5)	0.0108 (4)	0.0176 (5)	−0.0022 (4)	−0.0036 (4)	−0.0001 (3)
O5	0.0199 (5)	0.0182 (5)	0.0335 (6)	−0.0016 (4)	0.0032 (4)	−0.0038 (4)
N1	0.0153 (5)	0.0159 (5)	0.0194 (6)	−0.0022 (4)	−0.0036 (4)	0.0003 (4)
N2	0.0276 (6)	0.0118 (5)	0.0141 (5)	0.0027 (5)	−0.0032 (5)	−0.0016 (4)
C1	0.0137 (5)	0.0117 (6)	0.0144 (6)	−0.0022 (4)	−0.0020 (4)	−0.0027 (4)
C2	0.0170 (6)	0.0129 (6)	0.0146 (6)	−0.0027 (5)	−0.0039 (5)	0.0015 (4)
C3	0.0207 (6)	0.0137 (6)	0.0134 (6)	−0.0014 (5)	−0.0019 (5)	−0.0013 (4)
C4	0.0158 (6)	0.0120 (6)	0.0155 (6)	−0.0018 (5)	−0.0019 (5)	−0.0021 (4)
C5	0.0138 (5)	0.0108 (5)	0.0141 (6)	−0.0035 (5)	−0.0022 (4)	−0.0006 (4)
C6	0.0129 (5)	0.0128 (6)	0.0147 (6)	−0.0044 (5)	−0.0023 (4)	−0.0019 (4)
C7	0.0118 (5)	0.0137 (6)	0.0155 (6)	−0.0050 (5)	−0.0019 (4)	0.0002 (4)
C8	0.0317 (8)	0.0147 (6)	0.0144 (6)	0.0003 (6)	−0.0010 (5)	−0.0025 (5)
C9	0.0205 (6)	0.0153 (6)	0.0169 (6)	−0.0024 (5)	−0.0022 (5)	−0.0028 (5)
C10	0.0205 (6)	0.0173 (6)	0.0179 (6)	−0.0011 (5)	−0.0019 (5)	−0.0015 (5)
C11	0.0266 (7)	0.0137 (6)	0.0210 (7)	0.0011 (5)	−0.0064 (5)	−0.0033 (5)
C12	0.0252 (7)	0.0158 (7)	0.0351 (8)	−0.0059 (6)	−0.0027 (6)	−0.0037 (6)
Cl2	0.0479 (2)	0.02199 (18)	0.01606 (16)	−0.00506 (16)	−0.00745 (15)	−0.00001 (13)
O6	0.0176 (4)	0.0127 (4)	0.0196 (5)	0.0003 (4)	−0.0008 (4)	−0.0010 (4)
O7	0.0203 (5)	0.0165 (5)	0.0149 (4)	−0.0031 (4)	−0.0048 (4)	−0.0003 (3)
C13	0.0144 (6)	0.0130 (6)	0.0168 (6)	−0.0029 (5)	−0.0032 (5)	−0.0016 (4)
C14	0.0187 (6)	0.0114 (6)	0.0182 (6)	−0.0026 (5)	−0.0038 (5)	0.0002 (5)
C15	0.0268 (7)	0.0167 (6)	0.0146 (6)	−0.0067 (5)	−0.0036 (5)	0.0006 (5)
C16	0.0299 (7)	0.0166 (7)	0.0204 (7)	−0.0033 (6)	0.0017 (6)	−0.0047 (5)
C17	0.0282 (7)	0.0137 (6)	0.0257 (7)	0.0036 (6)	−0.0017 (6)	−0.0020 (5)
C18	0.0217 (7)	0.0156 (6)	0.0192 (6)	0.0000 (5)	−0.0031 (5)	0.0012 (5)
C19	0.0119 (5)	0.0137 (6)	0.0170 (6)	−0.0037 (5)	−0.0039 (4)	−0.0002 (4)
Na1	0.0119 (2)	0.0117 (2)	0.0173 (3)	−0.00179 (19)	−0.00343 (19)	−0.00038 (19)
Na2	0.0129 (2)	0.0116 (2)	0.0173 (3)	−0.00220 (19)	−0.00328 (19)	−0.00089 (19)
O8	0.0164 (4)	0.0126 (4)	0.0164 (5)	−0.0017 (4)	−0.0026 (4)	−0.0003 (4)
O9	0.0129 (4)	0.0153 (4)	0.0168 (5)	−0.0006 (4)	−0.0040 (4)	−0.0025 (3)
O10	0.0168 (4)	0.0128 (4)	0.0182 (5)	−0.0054 (4)	−0.0022 (4)	−0.0011 (4)
O11	0.0138 (4)	0.0139 (4)	0.0173 (5)	−0.0006 (4)	−0.0022 (4)	−0.0006 (4)
O12	0.0196 (5)	0.0178 (5)	0.0187 (5)	−0.0063 (4)	−0.0044 (4)	−0.0014 (4)
O13	0.0127 (4)	0.0158 (5)	0.0181 (5)	−0.0030 (4)	−0.0036 (4)	0.0012 (4)
O14	0.0142 (5)	0.0155 (5)	0.0207 (5)	−0.0006 (4)	−0.0008 (4)	−0.0025 (4)
O15	0.0168 (5)	0.0142 (5)	0.0205 (5)	−0.0047 (4)	−0.0065 (4)	−0.0005 (4)
O16	0.0138 (4)	0.0150 (5)	0.0184 (5)	−0.0041 (4)	−0.0020 (4)	−0.0031 (4)

### Geometric parameters (Å, º)

**Table:**

Cl1—C2	1.7411 (14)	C14—H14	0.9500
S1—O2	1.4418 (10)	C15—C16	1.391 (2)
S1—O1	1.4427 (10)	C16—C17	1.392 (2)
S1—N1	1.6120 (13)	C16—H16	0.9500
S1—C1	1.7604 (14)	C17—C18	1.393 (2)
O3—C7	1.2572 (16)	C17—H17	0.9500
O4—C7	1.2761 (16)	C18—H18	0.9500
O5—C9	1.3664 (17)	Na1—O14	2.3558 (13)
O5—C12	1.3703 (18)	Na1—O10	2.3946 (12)
N1—H11N	0.839 (18)	Na1—O9^i^	2.4090 (12)
N1—H12N	0.872 (18)	Na1—O9	2.4091 (13)
N2—C4	1.3512 (17)	Na1—O8	2.4134 (13)
N2—C8	1.4483 (18)	Na1—O11	2.4200 (12)
N2—H2N	0.860 (18)	Na2—O15	2.3709 (13)
C1—C6	1.3919 (18)	Na2—O16	2.3718 (13)
C1—C2	1.4001 (18)	Na2—O12	2.4016 (13)
C2—C3	1.3755 (19)	Na2—O13^ii^	2.4108 (12)
C3—C4	1.4163 (18)	Na2—O8	2.4294 (12)
C3—H3	0.9500	Na2—O13	2.4500 (13)
C4—C5	1.4295 (18)	O8—H81	0.83 (2)
C5—C6	1.3892 (18)	O8—H82	0.821 (19)
C5—C7	1.5066 (18)	O9—Na1^i^	2.4090 (12)
C6—H6	0.9500	O9—H91	0.862 (19)
C8—C9	1.4907 (19)	O9—H92	0.806 (19)
C8—H8A	0.9900	O10—H101	0.784 (19)
C8—H8B	0.9900	O10—H102	0.932 (18)
C9—C10	1.3501 (19)	O11—H111	0.842 (19)
C10—C11	1.436 (2)	O11—H112	0.811 (19)
C10—H10	0.9500	O12—H121	0.848 (19)
C11—C12	1.344 (2)	O12—H122	0.76 (2)
C11—H11	0.9500	O13—Na2^ii^	2.4108 (12)
C12—H12	0.9500	O13—H131	0.807 (19)
Cl2—C15	1.7510 (15)	O13—H132	0.848 (19)
O6—C19	1.2637 (16)	O14—H141	0.76 (2)
O7—C19	1.2623 (16)	O14—H142	0.74 (2)
C13—C18	1.3938 (19)	O15—H151	0.853 (19)
C13—C14	1.3995 (18)	O15—H152	0.803 (19)
C13—C19	1.5158 (19)	O16—H161	0.798 (19)
C14—C15	1.3857 (19)	O16—H162	0.80 (2)
			
O2—S1—O1	118.19 (6)	O7—C19—O6	124.65 (13)
O2—S1—N1	106.89 (7)	O7—C19—C13	118.41 (12)
O1—S1—N1	106.20 (7)	O6—C19—C13	116.94 (12)
O2—S1—C1	108.39 (6)	O14—Na1—O10	99.71 (4)
O1—S1—C1	106.54 (6)	O14—Na1—O9^i^	163.93 (4)
N1—S1—C1	110.57 (6)	O10—Na1—O9^i^	91.71 (4)
C9—O5—C12	106.30 (11)	O14—Na1—O9	103.75 (5)
S1—N1—H11N	112.4 (11)	O10—Na1—O9	81.57 (4)
S1—N1—H12N	112.1 (11)	O9^i^—Na1—O9	89.00 (4)
H11N—N1—H12N	116.8 (16)	O14—Na1—O8	77.59 (4)
C4—N2—C8	124.07 (12)	O10—Na1—O8	95.33 (4)
C4—N2—H2N	113.6 (12)	O9^i^—Na1—O8	90.19 (4)
C8—N2—H2N	121.6 (12)	O9—Na1—O8	176.78 (4)
C6—C1—C2	118.22 (12)	O14—Na1—O11	89.24 (4)
C6—C1—S1	118.85 (10)	O10—Na1—O11	159.01 (4)
C2—C1—S1	122.88 (10)	O9^i^—Na1—O11	83.87 (4)
C3—C2—C1	121.58 (12)	O9—Na1—O11	77.85 (4)
C3—C2—Cl1	117.22 (10)	O8—Na1—O11	105.17 (4)
C1—C2—Cl1	121.21 (10)	O15—Na2—O16	94.41 (4)
C2—C3—C4	120.46 (12)	O15—Na2—O12	99.60 (4)
C2—C3—H3	119.8	O16—Na2—O12	88.32 (4)
C4—C3—H3	119.8	O15—Na2—O13^ii^	96.08 (4)
N2—C4—C3	120.49 (12)	O16—Na2—O13^ii^	81.69 (4)
N2—C4—C5	121.12 (12)	O12—Na2—O13^ii^	162.00 (4)
C3—C4—C5	118.38 (12)	O15—Na2—O8	153.51 (4)
C6—C5—C4	119.11 (12)	O16—Na2—O8	111.98 (4)
C6—C5—C7	118.49 (11)	O12—Na2—O8	83.99 (4)
C4—C5—C7	122.30 (11)	O13^ii^—Na2—O8	85.91 (4)
C5—C6—C1	122.19 (12)	O15—Na2—O13	74.54 (4)
C5—C6—H6	118.9	O16—Na2—O13	166.06 (4)
C1—C6—H6	118.9	O12—Na2—O13	101.72 (4)
O3—C7—O4	123.35 (12)	O13^ii^—Na2—O13	90.90 (4)
O3—C7—C5	119.00 (11)	O8—Na2—O13	79.02 (4)
O4—C7—C5	117.62 (11)	Na1—O8—Na2	105.44 (4)
N2—C8—C9	110.13 (12)	Na1—O8—H81	118.9 (13)
N2—C8—H8A	109.6	Na2—O8—H81	106.7 (12)
C9—C8—H8A	109.6	Na1—O8—H82	116.0 (13)
N2—C8—H8B	109.6	Na2—O8—H82	102.7 (12)
C9—C8—H8B	109.6	H81—O8—H82	105.7 (17)
H8A—C8—H8B	108.1	Na1^i^—O9—Na1	91.00 (4)
C10—C9—O5	110.48 (12)	Na1^i^—O9—H91	105.6 (12)
C10—C9—C8	134.27 (13)	Na1—O9—H91	120.6 (12)
O5—C9—C8	115.26 (12)	Na1^i^—O9—H92	109.7 (13)
C9—C10—C11	106.27 (13)	Na1—O9—H92	118.4 (13)
C9—C10—H10	126.9	H91—O9—H92	108.7 (17)
C11—C10—H10	126.9	Na1—O10—H101	97.5 (13)
C12—C11—C10	106.22 (13)	Na1—O10—H102	122.0 (11)
C12—C11—H11	126.9	H101—O10—H102	105.4 (17)
C10—C11—H11	126.9	Na1—O11—H111	116.0 (11)
C11—C12—O5	110.72 (13)	Na1—O11—H112	104.5 (13)
C11—C12—H12	124.6	H111—O11—H112	105.9 (17)
O5—C12—H12	124.6	Na2—O12—H121	115.2 (13)
C18—C13—C14	119.61 (13)	Na2—O12—H122	135.5 (14)
C18—C13—C19	121.12 (12)	H121—O12—H122	103.8 (19)
C14—C13—C19	119.27 (12)	Na2^ii^—O13—Na2	89.10 (4)
C15—C14—C13	119.01 (12)	Na2^ii^—O13—H131	123.6 (13)
C15—C14—H14	120.5	Na2—O13—H131	111.3 (13)
C13—C14—H14	120.5	Na2^ii^—O13—H132	126.5 (12)
C14—C15—C16	122.04 (13)	Na2—O13—H132	106.2 (12)
C14—C15—Cl2	119.23 (11)	H131—O13—H132	98.4 (17)
C16—C15—Cl2	118.73 (11)	Na1—O14—H141	117.4 (14)
C15—C16—C17	118.54 (14)	Na1—O14—H142	113.1 (15)
C15—C16—H16	120.7	H141—O14—H142	104 (2)
C17—C16—H16	120.7	Na2—O15—H151	104.6 (12)
C16—C17—C18	120.34 (13)	Na2—O15—H152	119.8 (13)
C16—C17—H17	119.8	H151—O15—H152	106.5 (18)
C18—C17—H17	119.8	Na2—O16—H161	112.4 (13)
C17—C18—C13	120.46 (13)	Na2—O16—H162	117.0 (13)
C17—C18—H18	119.8	H161—O16—H162	106.8 (18)
C13—C18—H18	119.8		
			
O2—S1—C1—C6	119.77 (10)	C10—C11—C12—O5	0.73 (17)
O1—S1—C1—C6	−8.40 (12)	C9—O5—C12—C11	−0.22 (17)
N1—S1—C1—C6	−123.38 (11)	C18—C13—C14—C15	0.23 (19)
O2—S1—C1—C2	−57.59 (12)	C19—C13—C14—C15	179.71 (12)
O1—S1—C1—C2	174.23 (10)	C13—C14—C15—C16	−0.7 (2)
N1—S1—C1—C2	59.26 (13)	C13—C14—C15—Cl2	179.03 (10)
C6—C1—C2—C3	−0.01 (19)	C14—C15—C16—C17	0.5 (2)
S1—C1—C2—C3	177.37 (10)	Cl2—C15—C16—C17	−179.18 (12)
C6—C1—C2—Cl1	179.75 (10)	C15—C16—C17—C18	0.1 (2)
S1—C1—C2—Cl1	−2.87 (16)	C16—C17—C18—C13	−0.5 (2)
C1—C2—C3—C4	2.1 (2)	C14—C13—C18—C17	0.3 (2)
Cl1—C2—C3—C4	−177.66 (10)	C19—C13—C18—C17	−179.15 (13)
C8—N2—C4—C3	−10.5 (2)	C18—C13—C19—O7	−10.56 (19)
C8—N2—C4—C5	170.04 (13)	C14—C13—C19—O7	169.97 (11)
C2—C3—C4—N2	177.67 (12)	C18—C13—C19—O6	169.39 (12)
C2—C3—C4—C5	−2.87 (19)	C14—C13—C19—O6	−10.09 (18)
N2—C4—C5—C6	−178.95 (12)	O14—Na1—O8—Na2	117.64 (5)
C3—C4—C5—C6	1.59 (18)	O10—Na1—O8—Na2	−143.56 (4)
N2—C4—C5—C7	−2.64 (19)	O9^i^—Na1—O8—Na2	−51.83 (5)
C3—C4—C5—C7	177.90 (11)	O11—Na1—O8—Na2	31.87 (5)
C4—C5—C6—C1	0.48 (19)	Na1^i^—Na1—O8—Na2	−54.90 (7)
C7—C5—C6—C1	−175.97 (11)	O15—Na2—O8—Na1	−165.17 (8)
C2—C1—C6—C5	−1.29 (18)	O16—Na2—O8—Na1	20.07 (6)
S1—C1—C6—C5	−178.78 (10)	O12—Na2—O8—Na1	−65.67 (5)
C6—C5—C7—O3	−12.06 (17)	O13^ii^—Na2—O8—Na1	99.40 (5)
C4—C5—C7—O3	171.61 (11)	O13—Na2—O8—Na1	−168.88 (5)
C6—C5—C7—O4	166.10 (11)	Na2^ii^—Na2—O8—Na1	145.27 (4)
C4—C5—C7—O4	−10.23 (18)	O14—Na1—O9—Na1^i^	−170.12 (4)
C4—N2—C8—C9	−156.88 (13)	O10—Na1—O9—Na1^i^	91.87 (4)
C12—O5—C9—C10	−0.43 (16)	O9^i^—Na1—O9—Na1^i^	0.0
C12—O5—C9—C8	179.48 (12)	O11—Na1—O9—Na1^i^	−83.94 (4)
N2—C8—C9—C10	−119.97 (17)	O15—Na2—O13—Na2^ii^	96.06 (4)
N2—C8—C9—O5	60.15 (16)	O16—Na2—O13—Na2^ii^	57.56 (17)
O5—C9—C10—C11	0.86 (16)	O12—Na2—O13—Na2^ii^	−167.08 (4)
C8—C9—C10—C11	−179.02 (15)	O13^ii^—Na2—O13—Na2^ii^	0.0
C9—C10—C11—C12	−0.96 (16)	O8—Na2—O13—Na2^ii^	−85.65 (4)

Symmetry codes: (i) −*x*, −*y*+1, −*z*+1; (ii) −*x*−1, −*y*+2, −*z*+1.

### Hydrogen-bond geometry (Å, º)

**Table:**

*D*—H···*A*	*D*—H	H···*A*	*D*···*A*	*D*—H···*A*
N1—H11*N*···Cl1	0.839 (18)	2.767 (17)	3.2848 (16)	121.6 (14)
N1—H12*N*···O6	0.872 (18)	1.920 (18)	2.7905 (17)	175.3 (17)
N2—H2*N*···O4	0.860 (18)	1.906 (18)	2.6199 (16)	139.4 (16)
O8—H81···O3^iii^	0.83 (2)	2.07 (2)	2.8737 (16)	162 (2)
O8—H82···O15^ii^	0.82 (2)	2.00 (2)	2.8025 (16)	168 (2)
O9—H91···O16^i^	0.86 (2)	1.99 (2)	2.8499 (15)	172 (2)
O9—H92···O7	0.81 (2)	2.10 (2)	2.8867 (16)	167 (2)
O10—H101···O11^i^	0.78 (2)	1.96 (2)	2.7444 (16)	174 (2)
O10—H102···O3^iv^	0.93 (2)	1.90 (2)	2.8271 (15)	171 (2)
O11—H111···O6	0.84 (2)	1.89 (2)	2.7296 (16)	176 (2)
O11—H112···O12	0.81 (2)	2.08 (2)	2.8641 (16)	162 (2)
O12—H121···O4^iii^	0.85 (2)	1.96 (2)	2.7962 (16)	171 (2)
O12—H122···O2^v^	0.76 (2)	2.12 (2)	2.8593 (15)	165 (2)
O13—H131···O7^iii^	0.81 (2)	2.01 (2)	2.8082 (15)	169 (2)
O13—H132···O3^iii^	0.85 (2)	1.97 (2)	2.7939 (15)	165 (2)
O14—H141···O4^iii^	0.76 (2)	1.99 (2)	2.7265 (16)	164 (2)
O14—H142···O1	0.74 (2)	2.31 (2)	3.0019 (17)	156 (2)
O15—H151···O14^v^	0.85 (2)	1.92 (2)	2.7766 (16)	178 (2)
O15—H152···O7^iii^	0.80 (2)	2.14 (2)	2.8545 (16)	149 (2)
O16—H161···O10^v^	0.798 (19)	1.990 (19)	2.7857 (16)	175.7 (18)
O16—H162···O1^v^	0.80 (2)	2.20 (2)	2.9852 (15)	165.8 (17)

Symmetry codes: (i) −*x*, −*y*+1, −*z*+1; (ii) −*x*−1, −*y*+2, −*z*+1; (iii) *x*−1, *y*+1, *z*; (iv) −*x*+1, −*y*+1, −*z*+1; (v) *x*−1, *y*, *z*.
